# A Weak Response to Endoplasmic Reticulum Stress Is Associated With Postoperative Organ Failure in Patients Undergoing Cardiac Surgery With Cardiopulmonary Bypass

**DOI:** 10.3389/fmed.2020.613518

**Published:** 2021-02-15

**Authors:** Thomas Clavier, Zoé Demailly, Xavier Semaille, Caroline Thill, Jean Selim, Benoit Veber, Fabien Doguet, Vincent Richard, Emmanuel Besnier, Fabienne Tamion

**Affiliations:** ^1^Rouen University Hospital, Department of Anesthesiology and Critical Care, Rouen, France; ^2^Normandie Univ, UNIROUEN, INSERM U1096, FHU REMOD-VHF, Rouen, France; ^3^Rouen University Hospital, Department of Biostatistics, Rouen, France; ^4^Rouen University Hospital, Department of Cardiac Surgery, Rouen, France; ^5^Rouen University Hospital, Department of Medical Critical Care, Rouen, France

**Keywords:** bypass, cardiopulmonary, cardiac surgery, endoplasmic reticulum stress, endothelium, inflammation, GRP78 protein, human

## Abstract

**Introduction:** Endoplasmic reticulum stress (ERS) is involved in inflammatory organ failure. Our objective was to describe ERS, its unfolded protein response (UPR) expression/kinetics during cardiac surgery with cardiopulmonary bypass (CPB) and its association with postoperative organ failure (OF).

**Methods:** Prospective study conducted on patients undergoing cardiac surgery with CPB. Blood samples were taken before (Pre-CPB), 2 h (H2-CPB) and 24 h (H24-CPB) after CPB. Plasma levels of 78 kDa Glucose- Regulated Protein (GRP78, final effector of UPR) were evaluated by ELISA. The expression of genes coding for key elements of UPR (*ATF6, ATF4, sXBP1, CHOP*) was evaluated by quantitative PCR performed on total blood. OF was defined as invasive mechanical ventilation and/or acute kidney injury and/or hemodynamic failure requiring catecholamines.

**Results:** We included 46 patients, GRP78 was decreased at H2-CPB [1,328 (878–1,730) ng/ml vs. 2,348 (1,655–3,730) ng/ml Pre-CPB; *p* < 0.001] but returned to basal levels at H24-CPB [2,068 (1,436–3,005) ng/ml]. The genes involved in UPR had increased expression at H2 and H24. GRP78 plasma levels in patients with OF at H24-CPB (*n* = 10) remained below Pre-CPB levels [−27.6 (−51.5; −24.2)%] compared to patients without OF (*n* = 36) in whom GRP78 levels returned to basal levels [0.6 (−28.1; 26.6)%; *p* < 0.01]. H24-CPB *ATF6* and *CHOP* expressions were lower in patients with OF than in patients without OF [2.3 (1.3–3.1) vs. 3.0 (2.7–3.7), *p* < 0.05 and 1.3 (0.9–2.0) vs. 2.2 (1.7–2.9), *p* < 0.05, respectively].

**Conclusions:** Low relative levels of GRP78 and weak UPR gene expression appeared associated with postoperative OF. Further studies are needed to understand ERS implication during acute organ failure in humans.

## Introduction

Cardio-pulmonary bypass (CPB) is routinely used throughout the world during heart surgery. This procedure often induces an aseptic systemic inflammatory response syndrome (SIRS) associated with post-operative morbidity (1–3). This SIRS might lead to hypotension and organ dysfunction, a situation referred to as “post-pump syndrome” (4). Given the association between elevated pro-inflammatory cytokine levels and negative clinical outcomes [post-operative acute kidney injury (AKI), decreased systemic vascular resistance and lung injury], it has been postulated that modulation of inflammatory processes could improve outcomes after cardiac surgery (2, 5). Despite a progression in knowledge of CPB-induced SIRS pathophysiology, specific therapeutics to control inflammatory process are still lacking.

Endoplasmic reticulum (ER) stress and its adaptive response, the unfolded protein response (UPR), represent an archetypal example of adaptive stress responses. The ER plays a crucial role in protein folding and maturation. This folding process is finely regulated, notably by specific proteins known as chaperones, such as the 78 kDa Glucose-Regulated Protein [GRP78, a heat-shock protein coded by the Heat Shock 70kDa Protein 5 gene (*HSPA5*)], which stimulates the correct folding of polypeptide to functional protein complexes (6). Multiple disturbances observed during inflammation can result in a dysfunction of the ER, leading to the accumulation of unfolded proteins within the lumen of the ER, known as ER stress (ERS) (6, 7). The defense against ERS mainly involves the UPR which relies on three signaling pathways: Inositol-Requiring Protein-1 alpha pathway [IRE1α, involving the spliced ribonucleic acid (RNA) of X-box binding protein 1 (sXBP1)], Protein Kinase RNA-like ER kinase pathway [PERK, involving CCAAT/enhancer binding protein homologous protein (CHOP) and Activating Transcription Factor 4 (ATF4)] and Activating Transcription Factor 6 (ATF6) pathway (6). One of the roles of the UPR is to lead the synthesis of new chaperones to allow protein folding (e.g., GRP78, a final effector of UPR). However, if the ERS is severe and prolonged, UPR can lead to cell death by apoptosis (8).

Cytokine synthesis induces a massive increase in protein synthesis and, thus, an ERS which in turn activates NF-kB and thus maintains this synthesis (9). Cellular dysfunction, hallmarked by ERS, is increasingly recognized as an important contributor to the development of organ failure in critical illness, and in particular during systemic inflammation (6, 10, 11). ERS induces dysfunction and apoptosis of cardiomyocytes that can lead to heart failure and UPR have a protective effect on acute or chronic heart failure (12). ERS is associated with endothelial dysfunction and its inhibition improves endothelium-dependent relaxing function (13). In experimental sepsis, a treatment with 4-phenylbutyric acid (4BPA; a chemical chaperone which inhibits ERS) decreases the tissue expression level of inflammatory cytokines, reduces organ dysfunction and improves survival (14, 15). In human, ERS is activated in the mononuclear cells of patients with septic acute lung injury, is involved in AKI and its expression is partly correlated with organ failure in patients with septic shock (10, 14, 16). A recent work has shown the feasibility of the non-invasive detection of the ERS in urine in patients undergoing cardiac surgery with CPB and indicates that an early and robust adaptive UPR is critical for endogenous protection to acute renal failure (17).

Thus, we designed a prospective study to describe the kinetics of UPR markers and to evaluate the link between UPR expression and organ failure in patients undergoing elective cardiac surgery with CPB.

## Materials and Methods

### Study Design

This prospective pilot study was conducted in the cardiac surgery ICU of a tertiary care University Hospital between July 2018 and April 2019. The study (N°2017/179/HP) was approved by the ethics committee *Sud-Méditerrannée II* (n° CPP 2017-A03375-48) and was performed in accordance with French laws and with the ethical standards laid down in the Declaration of Helsinki and its later amendments (18).

### Inclusion and Exclusion Criteria

Adult patients (≥18 y/o) who underwent cardiac surgery with an estimated duration of CPB of more than 1 h were eligible to be included in the study. Eligible patients were contacted, and written informed consent was obtained prior to inclusion.

Exclusion criteria were: age under 18 y/o or patient under guardianship, pregnancy/breastfeeding, urgent surgery, predictable CPB of <1 h [single or double coronary artery bypass grafting (CABG) or single aortic valve replacement (VR)], surgery without sternotomy, a history of altered left ventricular systolic function (<30%), chronic autoimmune inflammatory disease, neoplasia.

### Objectives

#### Primary Objective

The primary objective was to evaluate the variation in GRP78 plasma levels before CPB (Pre-CPB) and 2 and 24 h after the end of CPB.

#### Secondary Objectives

Secondary objectives were to evaluate:

- the kinetics of UPR pathway gene (*ATF6, ATF4, CHOP, HSPA5*, and *sXBP1*) expression in whole blood after CPB;
- the association between GRP78 plasma level variations and endothelial dysfunction markers [Syndecan-1, Vascular Cell Adhesion Protein 1 (VCAM-1)], or inflammatory cytokine interleukin (IL)-6;
- the association between GRP78 level variations or UPR gene expression and organ failure 24 h after CPB (defined as presence of: invasive mechanical ventilation and/or AKI (Kidney Disease Improving Global Outcomes score ≥ 1) and/or hemodynamic failure requiring catecholamines) by comparing two groups: patients with and patients without organ failure 24 h after CPB.

### Sample Collection and Analysis

#### Surgical Procedure

Induction of anesthesia was achieved with intravenous hypnotic (propofol or etomidate), opioid (sufentanil or remifentanil) and curare (cisatracrium). The anesthesia was maintained with propofol and continuous infusion of opioids. CPB was initiated with a heparinized solution. Oxygenated blood was re-injected into the arterial circulation through a cannula inserted into the aorta downstream of aortic clamping. The heart was stopped by infusion of a cardioplegia solution (potassium and beta-blockers or Custodiol^©^ cardioplegia). During surgery, mean blood pressure was maintained between 55 and 70 mmHg. At the end of the procedure, circulating heparin was neutralized with protamine. Vasoconstrictors or inotropic agents, fluids, and transfusion products were administered at the discretion of the anesthesiologist based on clinical, echocardiographic and biological findings. Patients were transferred to post-operative cardiac ICU and monitored hourly for the first 24 h and then every 3 h for the remaining period of the ICU stay.

For each patient, baseline pre-operative characteristics were evaluated (sex, age, body mass index). The data relevant to the undertaken surgical procedure (type of surgery, surgery/CPB duration) and ICU stay [Simplified Acute Physiology Score (SAPS) II], use of catecholamine, duration of mechanical ventilation, length of ICU stay were collected.

#### Blood Sampling

All samples were collected from patients' arterial line, using standard hygiene protocols. The Pre-CPB sample was taken after induction of anesthesia and before incision, just after the arterial catheter was placed, postoperative samples were taken 2 and 24 h after the end of CPB, respectively. At each time point, one PAXgene® tube [allowing the conservation of ribonucleic acid (RNA) of circulating blood cells; Quiagen, Hilden, Germany; 2.5 ml of blood] and one EDTA tube (4 ml of blood) were collected. The EDTA tube was immediately centrifuged at 3,000 g for 15 min and plasma was aliquoted in microtubes. Samples were kept for a maximum of 7 days in the freezer of the ICU at −20°C and then were stored at −80°C until final analysis.

#### Enzyme Linked Immunosorbent Assay (ELISA)

Plasma GRP78 concentrations were determined using the commercial kit GRP78/BiP ADI-900-214 (Enzo Life Sciences, France) according to the manufacturer's protocol. After preliminary analyses, a dilution of our samples to 1:10 was chosen for optimized results. Other protein concentrations were determined using the Thermo Fisher Scientific (MA, USA) commercials kits IL-6 (ref. EH2IL6), Syndecan-1 (ref. EHSDC1) and VCAM-1 (ref. KHT0601) according to the manufacturer's protocol.

#### Ribonucleic Acid Extraction, Reverse Transcription, and Quantitative Polymerase Chain Reaction

RNA extraction was performed using the commercial kit PAXgene® Blood RNA System kit (Quiagen, Hilden, Germany) according to the manufacturer's protocol. Before RNA elution, residual genomic deoxyribonucleic acid (DNA) was digested using RNase-Free DNase set (Quiagen, Hilden, Germany). The integrity and quantity of the total RNA were assessed with a Nanodrop 2000 device (Thermo Fisher Scientific, Waltham, MS, USA). Total RNAs were reverse transcribed into cDNA using M-MLV Reverse Transcriptase (Invitrogen, Carlsbad, CA, USA) according to manufacturer's instructions.

A quantitative polymerase chain reaction (qPCR) was performed for:

- The mRNA of genes coding for proteins involved in UPR: *ATF6, ATF4, sXBP1, CHOP, HSPA5*;
- The mRNA of the gene coding for succinate dehydrogenase complex flavoprotein subunit A (SDHA). As *SDHA* has been described as a pertinent housekeeping gene in humans with inflammation and as its cycle threshold (Ct) is close to the Ct of UPR genes in qPCR, it appeared as the best housekeeping gene for our work (19).

The genes that were amplified and the primers that were used are listed in Table 1. Quantitative PCR was performed using the Quantstudio 12K Flex system (Applied Biosystems, Foster City, CA, USA) according to the manufacturer's instructions. The 384-well PCR plates were prepared with 1.2 μL of cDNA (16.7 ng/μL) diluted at 1:10 and 3.81 μL of reaction mix. The reaction mix contained the sense and antisense primers at a concentration of 300 nM (0.02 μL ×2), Fast Sybr Master mix (2.50 μL) and water DNase and RNase free (1.27 μL). The final volume was 5 μL per well. Samples were deposited using the Bravo Automated Liquid Handling Platform pipetting robot (Agilent Technologies, Santa Clara, CA, USA). The analysis included a first activation step for 20 s and then 40 amplification cycles consisting of a new activation phase at 95°C for 1 s followed by an elongation phase at 60°C for 20 s. Ct values were used for quantifying target gene expression relative to the housekeeping gene using the 2^−ΔΔCt^ method.

**Table 1:** Primers used for quantitative PCR.

**Gene name**	**Sense**	**Sequence (5^**′**^-3^**′**^)**
*SDHA*	Forward	GAGATGTGGTGTCTCGGTCCAT
	Reverse	GCTGTCTCTGAAATGCCAGGCA
*ATF4*	Forward	GTTCTCCAGCGACAAGGCTA
	Reverse	ATCCTGCTTGCTGTTGTTGG
*CHOP*	Forward	TCGCCGAGCTCTGATTGAC
	Reverse	CCCTGCGTATGTGGGATTGAG
*sXBP1*	Forward	TGCTGAGTCCGCAGCAGGTG
	Reverse	GCTGGCAGGCTCTGGGGAAG
*ATF6*	Forward	CCGCAGAAGGGGAGACACA
	Reverse	TCGGAGGTAAGGAGGAACTGACG
*HSPA5*	Forward	CGAGGAGGAGGACAAGAAGG
	Reverse	CACCTTGAACGGCAAGAACT

*ATF4/6, Activating Transcription Factor 4/6; CHOP, CCAAT/enhancer binding protein homologous protein; HSPA5, Heat Shock 70kDa Protein 5; SDHA, succinate dehydrogenase complex flavoprotein subunit A; sXBP1, spliced X-box binding protein 1*.

### Statistical Analysis

In view of a previous work studying UPR expression during septic SIRS, we considered that it was necessary to include at least 45 patients to highlight a significant UPR after CPB (10). Since each subject was taken as its own control, the non-parametric Wilcoxon matched pairs signed rank test was used to assess significant variations in quantitative parameters. For group comparisons, the quantitative variables were compared using a Mann-Whitney test or a Student's test depending on the distribution of the data. The Pearson correlation test was used to assess the strength of the association between two quantitative variables. Continuous data are expressed as median with interquartile range, categorical data are presented as absolute values with percentages. All statistical tests were two-sided and the 0.05 probability level was used to establish statistical significance. The statistical analyses were performed by means of the statistical software SAS (version 9.4; SAS Institute; Cary, NC). The data were exported to GraphPad Prism 8.0 software for figure creation.

## Results

### Clinical and Demographic Characteristics of Population

Forty-six patients were enrolled between July 2018 and April 2019. Baseline and peri-operative characteristics of included patients are detailed in Table 2. All patients were alive at D28.

**Table 2:** Main demographic and clinical characteristics of patients.

**Demographic characteristics**	
Number of patients	46
Age (years)	70 (63–76)
Sex-ratio (M/F)	3.6 (36/10)
Body Mass Index (kg/m^2^)	28.1 (25.7–30.5)
Length of stay in hospital (days)	13 (9–17)
**Surgical characteristics**	
Type of surgery:	
- coronary artery bypass grafting (CABG)	9 (19.6 %)
- mitral valve surgery	10 (21.7 %)
- Bentall or Tirone-David surgery	10 (21.7 %)
- Ross surgery	1 (2.2 %)
- aortic + mitral valve surgery	5 (10.9 %)
- aortic valve surgery + CABG	5 (10.9 %)
- mitral valve surgery + CABG	4 (8.7 %)
- aortic + mitral valve surgery + CABG	2 (4.3 %)
Duration of surgery (min)	221 (186–254)
Duration of CPB (min)	117 (92–139)
**Hematocrit (%)**	
- before CPB	42 (26–49)
- 2 h after CPB	35 (27–40)
- 24 h after CPB	34 (26–41)
**ICU stay characteristics**	
SAPS II	33 (30–40)
Duration of mechanical ventilation (hours)	6 (4–8)
Length of ICU stay (days)	3 (2–5)

*Data are presented as median with interquartile range or absolute value and percentage [n (%)]. CABG, coronary artery bypass grafting; CPB, Cardiopulmonary Bypass; ICU, Intensive Care Unit; SAPS II, Simplified Acute Physiology Score II*.

### GRP78 Plasma Levels

**Figure 1:**
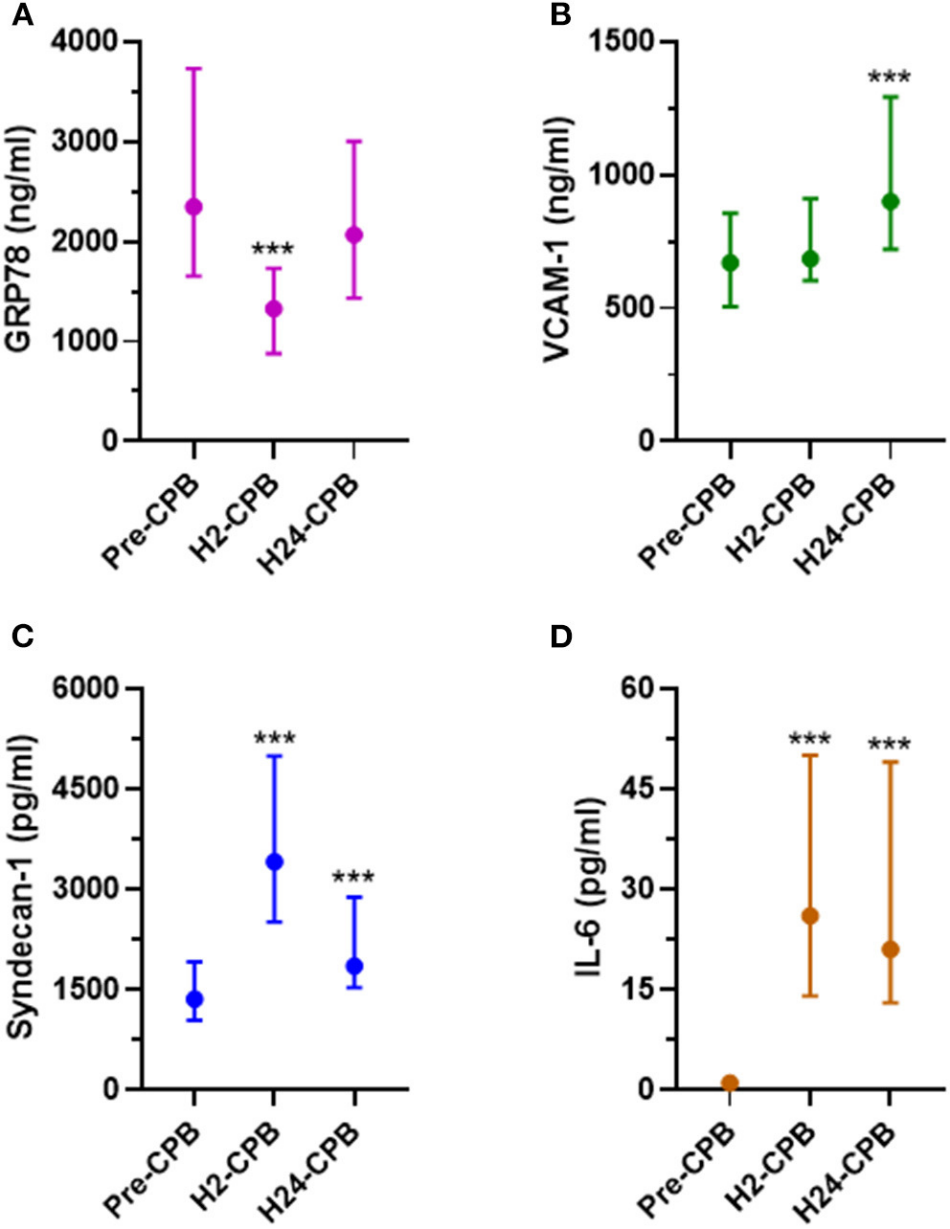
Plasma levels of GRP78 **(A)**, VCAM-1 **(B)**, Syndecan-1 **(C)** and IL-6 **(D)** before (Pre-CPB), 2 (H2-CPB) and 24 (H24-CPB) hours after cardiopulmonary bypass (CPB). The results show a post-operative increase in IL-6, Syndecan-1 and VCAM-1 and a transient decrease (at H2-CPB only) in GRP78. Dosages were performed by Enzyme linked immunosorbent assay (ELISA). Values are presented as median with interquartile range. ***p < 0.001 in comparison with Pre-CPB level. GRP78, 78 kDa Glucose-Regulated Protein; IL-6, Interleukin 6; VCAM-1, Vascular Cell Adhesion Protein 1.

**Figure 2:**
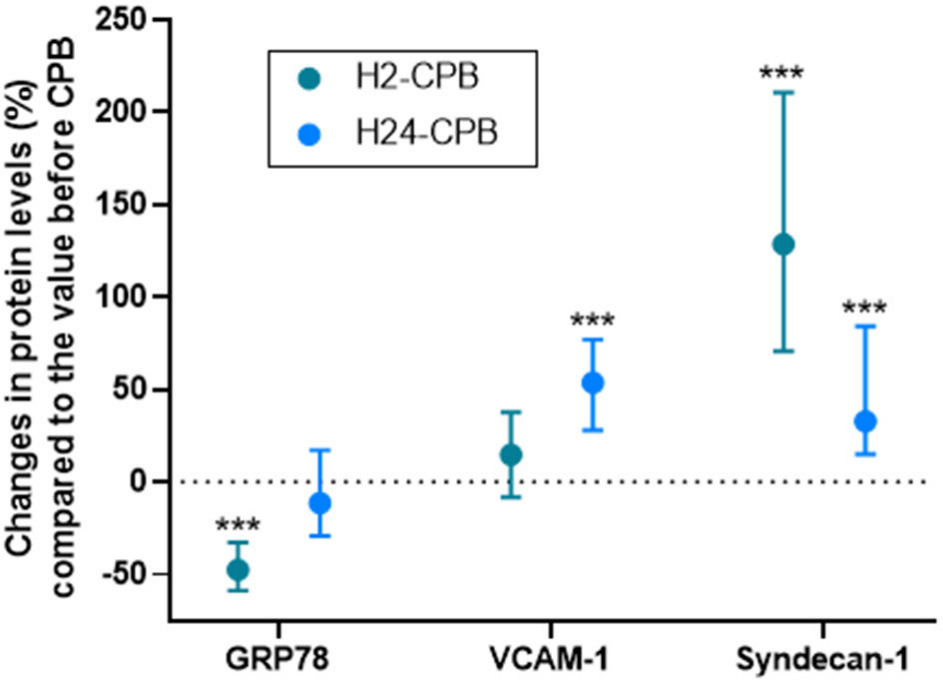
Relative levels of studied protein 2 (H2-CPB) and 24 (H24-CPB) hours after cardiopulmonary bypass (CPB). The results show a post-operative relative increase in Syndecan-1 and VCAM-1 and a transient decrease (2 h after CPB only) in GRP78. Dosages were performed by Enzyme linked immunosorbent assay (ELISA). Values are presented as median with interquartile range. ***p < 0.001 in comparison with Pre-CPB level. GRP78, 78 kDa Glucose-Regulated Protein; VCAM-1, Vascular Cell Adhesion Protein 1.

The plasma level of GRP78 was significantly decreased 2 h after CPB but there was no difference in GRP78 levels between pre-operative and 24-h post-CPB samples (Figure 1A). Relative changes in GRP78 levels 2 and 24 h after CPB are shown in Figure 2. There was no correlation between relative changes in GRP78 plasma level variation at 24 h and duration of CPB [*r* = −0.19 (−0.45; 0.11); *p* = 0.22]. There was no correlation between plasma level of C-reactive protein and plasma level of GRP78 at H24 [*r* = 0.17 (−0.13; 0.44); *p* = 0.26].

### Syndecan-1, VCAM-1, and IL-6 Plasma Levels

The plasma level of VCAM-1 did not show any change at 2 h after CPB but was significantly increased 24 h after CPB (Figure 1B) while syndecan-1 and IL-6 levels were increased 2 and 24 h after CPB (Figures 1C,D). Relative changes in studied protein levels at 2 and 24 h post-CPB (compared to the value before CPB) are shown in Figure 2 (as Pre-CPB IL-6 levels were undetectable, it was not possible to perform a relative analysis for this cytokine). Twenty-four hours after CPB, there was no correlation between GRP78 plasma level variations and VCAM-1 [*r* = 0.04 (−0.33; 0.25); *p* = 0.79] and Syndecan-1 [−0.29 (−0.53; 0.00); *p* = 0.05] plasma level variations or IL-6 absolute values [*r* = 0.12 (−0.18; 0.40); *p* = 0.43].

There was also no correlation of the absolute values of GRP78 rates with those of IL-6, VCAM-1, and Syndecan-1 at H24 (Supplemental Figure 1).

### Gene Expression of Unfolded Protein Response

**Figure 3:**
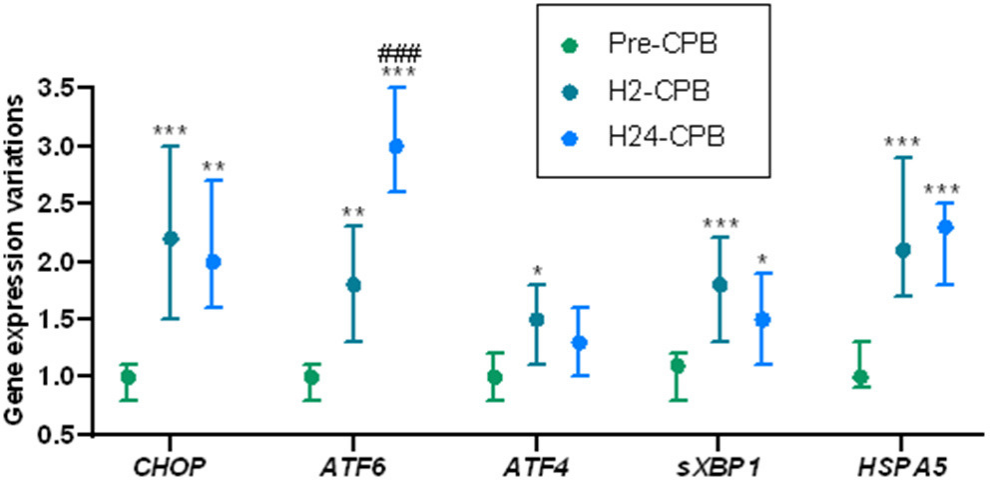
Relative changes in Unfolded Protein Response gene expression after cardiac surgery with cardiopulmonary bypass (CPB). The results show a post-operative increase in the expression of genes coding for the key proteins of unfolded protein response, meaning a postoperative activation of the unfolded protein response. Analyses were performed by quantitative polymerase chain reaction. Gene expression prior to CPB (Pre-CPB) was averaged to 1 for each gene. For each patient, expressions at 2 h (H2-CPB) and 24 h (H24-CPB) after CPB were expressed as relative to Pre-CPB. Values are presented as median with interquartile range. Differences expressed in comparison with preoperative gene expressions: *p < 0.05; **p < 0.01; ***p < 0.001. Differences expressed in comparison with H2-CPB gene expressions: ^*###*^*p* < 0.001. ATF, Activating Transcription Factor; CHOP, CCAAT/enhancer binding protein homologous protein; HSPA5, Heat Shock 70kDa Protein 5; sXBP1, spliced RNA of X-box binding protein 1.

The expression of *CHOP* and *sXBP1* was increased 2 h after CPB and remained stable 24 h after CPB. *ATF4* showed a small increase in expression 2 h after CPB but there was no difference in its expression between pre-operative and 24-h post-CPB samples (Figure 3). The expression of *ATF6* was increased 2 h after CPB and kept increasing 24 h after CPB (Figure 3).

### Correlation Between Unfolded Protein Response and Clinical Outcome

**Figure 4:**
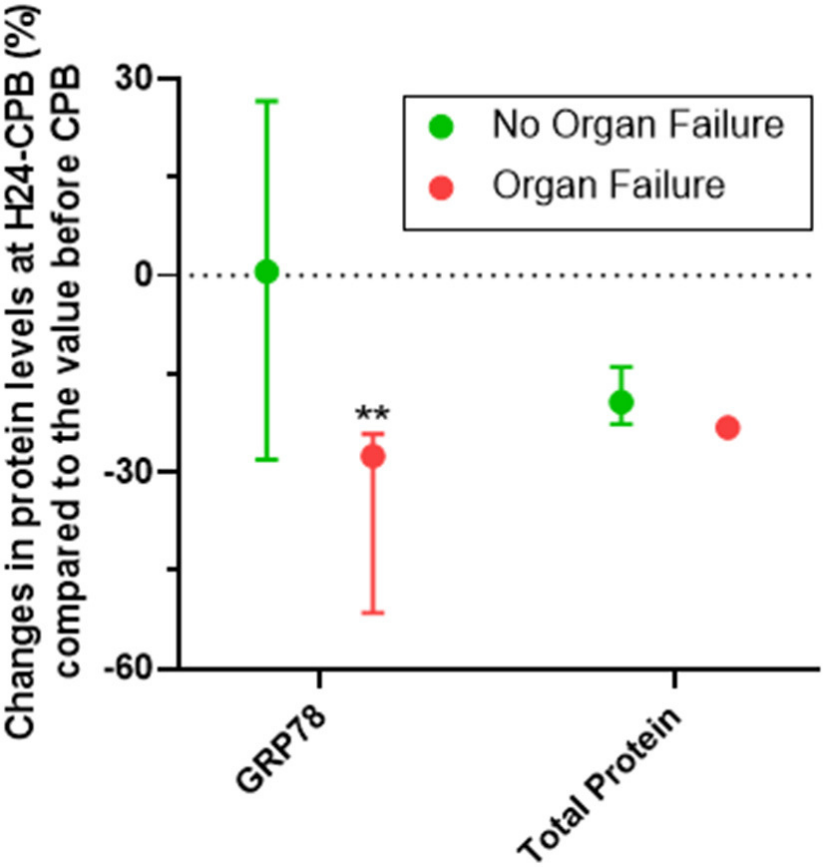
Relative changes (%) in GRP78 levels at 24 h after cardiopulmonary bypass (CPB; H24-CPB) in patients with or without organ failure. The results show that GRP78 levels remain below baseline in patients with organ failure while they return to baseline in patients without organ failure (with no difference in proteinemia between the two groups), suggesting a less intense unfolded protein response in patients with organ failure. Dosages were performed by Enzyme linked immunosorbent assay (ELISA). Values are presented as median with interquartile range. **p < 0.01 between groups. GRP78, 78 kDa Glucose-Regulated Protein.

**Figure 5:**
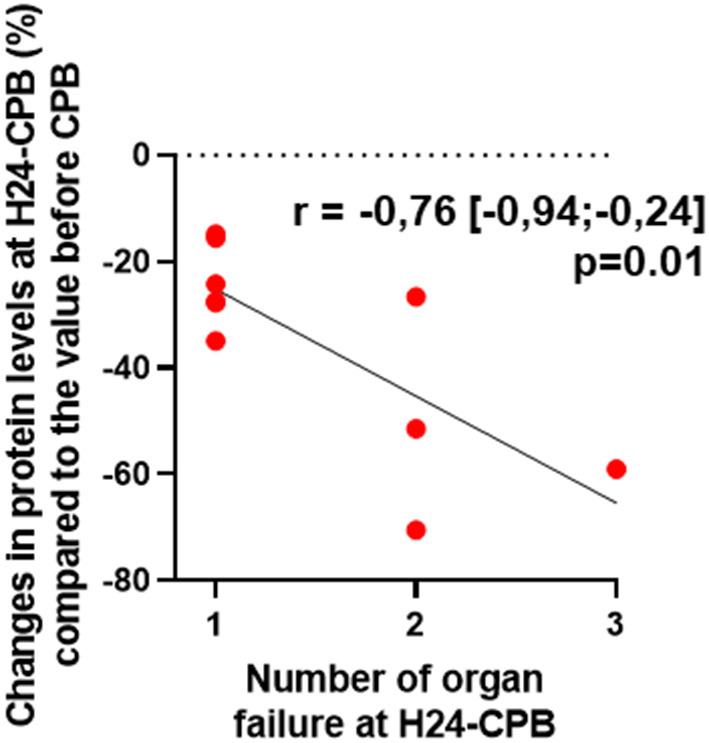
Correlation between relative changes (%) in GRP78 levels at 24 h after cardiopulmonary bypass (CPB; H24-CPB) and the number of organ failures in the subgroup of patients with organ failure. The results show that a high number of organ failure is correlated with a significant decrease in GRP78 levels compared to the baseline level before CPB. GRP78, 78 kDa Glucose-Regulated Protein.

**Figure 6:**
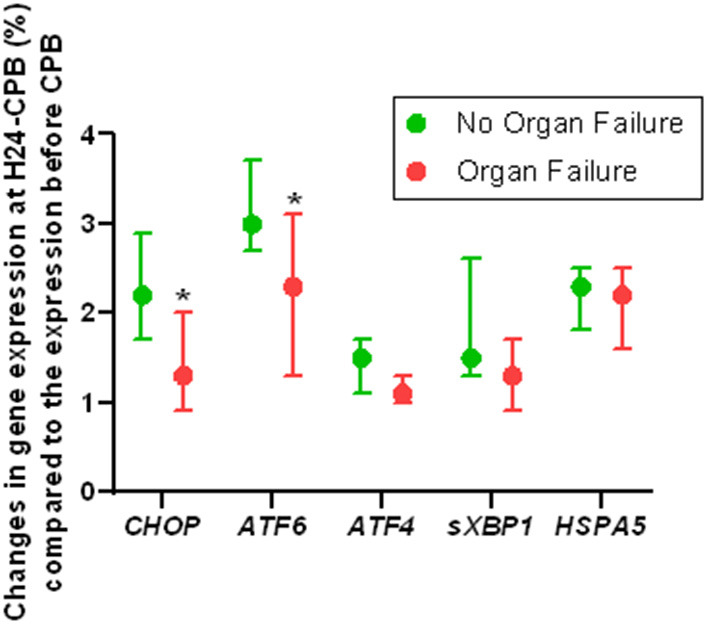
Relative changes in Unfolded Protein Response gene expression 24 h after cardiopulmonary bypass (CPB) in patients with or without organ failure. Results show that the expression of *ATF6* and *CHOP* genes (which code for unfolded protein response key proteins) is lower in patients with postoperative organ failure than in those without organ failure, suggesting a less intense unfolded protein response in patients with organ failure. Analyses were performed by quantitative polymerase chain reaction. Values are presented as median with interquartile range. *p < 0.05 between groups. Gene expression at 24 h after CPB was relative to preoperative expression. ATF, Activating Transcription Factor; CHOP, CCAAT/enhancer binding protein homologous protein; HSPA5, Heat Shock 70kDa Protein 5; sXBP1, spliced RNA of X-box binding protein 1.

Of the 46 patients, 10 had persistent organ failure 24 h after CPB (9 treated with catecholamines, 4 mechanically ventilated and 2 with acute renal failure; Supplementary Table 1). Their demographical and clinical characteristics are presented in the Table 3. There was no difference concerning Pre-CPB GRP78 levels and UPR gene expression between patients with or without organ failure (Supplementary Table 2). GRP78 plasma levels at 24-h post-CPB in patients with persistent organ failure remained significantly below Pre-CPB levels compared to patients without organ failure in whom GRP78 levels returned to baseline levels (Figure 4). To evaluate the potential bias induced by hemodilution on GRP78 levels according to the presence or absence of organ failure, we analyzed the variations in total protein levels between patients with and without organ failure and found no significant differences between groups (Figure 4). Among the patients with organ failure, the decrease in GRP78 levels between Pre-CPB and H24-CPB was correlated to the number of organ failures [*r* = −0.76 (−0.94 to −0.24); *p* = 0.01; Figure 5]. *ATF6* and *CHOP* expressions were significantly lower 24 h after CPB in patients with organ failure while there was no difference concerning *sXBP1* and *ATF4* expression (Figure 6).

**Table 3:** Comparison of demographic and clinical characteristics of patients with and without organ failure 24 h after cardiopulmonary bypass.

	**No organ failure** **(*n* = 36)**	**Organ failure** **(*n* = 10)**	***p***
**Demographic characteristics**			
Age (years)	71 (66–75)	69 (51–76)	0.46
Sex-ratio (M/F)	6.2	1.0	0.27
Body Mass Index (kg/m^2^)	27.0 (24.8–29.3)	31.5 (28.3–34.7)	0.02
Length of stay in hospital (days)	13 (9–16)	19 (11–24)	0.08
**Surgical characteristics**			
Type of surgery [*n* (%)]:			
- coronary artery bypass grafting (CABG)	8 (22%)	1 (10%)	0.79
- valve surgery	20 (56%)	6 (60%)	
- valve + CABG surgery	8 (22%)	3 (30%)	
Duration of surgery (min)	212 (179–254)	251 (208–260)	0.12
Duration of CPB (min)	109 (63–198)	138 (81–259)	0.70
**ICU stay characteristics**			
SAPS II	33 (27–38)	41 (31–49)	0.78
Duration of mechanical ventilation (hours)	5 (4–7)	11 (7–33)	0.02
Length of ICU stay (days)	3 (2–4)	5 (4–6)	0.08

*Data are presented as median with interquartile range or absolute value and percentage [n (%)]. CABG, coronary artery bypass grafting; CPB, Cardiopulmonary Bypass; ICU, Intensive Care Unit; SAPS II, Simplified Acute Physiology Score II*.

## Discussion

To our knowledge, we describe for the first time the kinetics of all UPR pathways to restore ER homeostasis in patients undergoing elective cardiac surgery with CPB. We found that the plasma level of GRP78, one of the final effectors of the UPR, was decreased at the initial phase of CPB-induced SIRS and that a persistent decrease in GRP78 levels was associated with postoperative organ failure in this population.

### Kinetics of Unfolded Protein Response

The present study demonstrates that CPB stimulated UPR, as reflected by the increased gene expression of the three UPR pathways: IRE1α, PERK, and ATF6.We observed a brief decrease in circulating GRP78 levels 2 h after CPB and a return to baseline level 24 h after CPB. We didn't find any correlation between changes in GRP78 at 24 h and duration of CPB but our range of CPB times was not wide. Future investigations should examine a broader range of CPB times before really concluding on this point. It is known that heat-shock proteins can be secreted extracellularly by many cells (dendritic cells, hepatocytes, myocytes, gut cells, lymphocytes, etc.) through several regulated pathways: lysosome-endosome pathway, secretory-like granules, extracellular vesicles (20). A previous work reported that extracellular GRP78 is mostly due to an active release from intact cells and does not result solely from the leakage of proteins from dead cells (21). It is therefore likely that the decrease in GRP78 plasma level is the result of an adaptive cellular mechanism.

Several studies have shown an increase in GRP78 plasma levels in patients with chronic systemic inflammation (cancer, obesity, atherosclerosis) (22–24). However, in acute systemic inflammation, there is increased demand for intracellular GRP78 to resolve the ERS (6, 25). This may explain the observed early decrease of extracellular GRP78 which is associated with a rapid activation of UPR gene transcription 2 h after CPB, allowing a return of GRP78 to baseline level 24 h after CPB. It appears normal for the transcription to precede the translation and it should be noted that the transcription of *HSPA5*, which codes for GRP78, is elevated early (from H2). However, the effective recovery of GRP78 plasma levels is only visualized at H24. This suggests that the activation of UPR genes is very rapid after inflammation but that its protein response in plasma is time-shifted. It has been shown that the UPR genes expression of each UPR pathway are highly correlated during ERS with variations among individual (26, 27). Our results show that the three pathways of UPR are activated during aseptic systemic inflammation in humans. However, the kinetics of these pathways appears to be different. The IRE1α (explored by *sXBP1*) and PERK (explored by *ATF4* and *CHOP*) pathways seemed to have a stable level of expression between 2 and 24 h after CPB. On the contrary, *ATF6* expression increased significantly 2 h after CPB and kept increasing 24 h after CPB. During ERS, ATF6 pathway is the first to be initiated and, due to its rapid activation (proteolytic cleavage and direct action on the genome as a transcription factor), it has the most reactive kinetics of the three UPR pathways (28, 29). ATF6 upregulates many protective genes and downregulates many potentially damaging genes, and previous studies have shown that ATF6 activation in cardiac myocytes protects the heart from ischemic damage, while inhibiting ATF6 has the opposite effect (30–32). Given our results and as previously suggested, it is possible that among UPR pathways, ATF6 is the most intensely involved pathway during acute SIRS (which could explain why its activation continued to increase 24 h after CPB) (10). The three UPR pathways have, in part, common effects to resolve ERS: chaperone synthesis, activation of ER associated degradation, activation of Nuclear Factor-Kappa B, etc. (6, 8). It is therefore difficult to propose a hypothesis on the clinical consequences of differential activation of the three pathways of UPR over time. Future works studying the activation kinetics of the UPR pathways in humans are in any case necessary to confirm or invalidate our results and to analyze more precisely the activation/return to normal delays after acute inflammation.

### Unfolded Protein Response and Organ Failure

It is known that apoptosis, cytokine release and oxidative stress induced by ERS can lead to organ failure during sepsis (15). To respond to ERS, cells activate an adaptative pathway, the UPR, to synthesize chaperones (including GRP78) and restore normal ER function. It can therefore be assumed that ERS after cardiac surgery can also be a source of organ failure. Circulating GRP78 levels returned to levels comparable to baseline at H24-CPB except in patients with persistent organ failure who maintained GRP78 levels below their initial baseline. They also had a lower UPR gene expression than patients without organ failure. In a previous study, the expression of UPR mRNA gene in urine after cardiac surgery showed that patients with a rapid increase in sXBP1 mRNAs expression in urine (reflecting kidney UPR) had less AKI (17). These data suggest that a robust post-operative activation of the UPR after CPB is critical for protecting against organ failure. Moreover, pre-clinical data show that the resolution of ERS *via* chemical chaperones (e.g., 4BPA) can correct organ failure induced by a septic SIRS (14, 15). In our study, patients with a relatively strong UPR response (that probably allowed ERS resolution) returned to their baseline chaperone levels with no organ failure, while patients with a weaker UPR response failed to return to their baseline chaperone levels and had persistent organ failure. Our results are therefore consistent with previous data in human and animals on the impact of ERS and UPR on organ failure during SIRS. But, as previously stated, the prognostic value of the markers of ERS response may change with the duration of adaptive responses, which also reflect the duration of the stress (17). While a strong UPR appears necessary in the acute stress phase, excessively prolonged ERS responses promote cell death as a result of an imbalance in favor of proapoptotic pathways rather than antiapoptotic pathways (8).

Extracellular GRP78 is known to have anti-inflammatory properties by inducing the endocytosis of the Toll-Like Receptor 4, reducing the production of inflammatory cytokines and increasing the synthesis of anti-inflammatory cytokines (33, 34). It is therefore possible that patients returning to pre-operative levels of GRP78 may also benefit from its immunomodulatory effect and thus be less likely to develop persistent organ failure than patients remaining at relatively low levels of extracellular GRP78. Nevertheless, our work does not establish a causal link between organ failure after CPB and the level of GRP78, and further works are therefore necessary to confirm or invalidate our observations.

### Syndecan-1, VCAM-1, and IL-6 Plasma Levels

It has been shown that ERS is implicated in endothelial dysfunction and that its inhibition in humans improves endothelial dysfunction induced by glucose ingestion (13, 35). Moreover, in a previous study conducted in septic patients, we have shown an association between expressions of *ATF6* and *ET1* (coding for endothelin-1 which is associated with endothelial dysfunction) (10). However, we did not find a link between VCAM-1, Syndecan-1 or IL-6 and GRP78 variations. With regard to the results of previous studies on the link between ERS and endothelial dysfunction, we should be cautious and not conclude the absence of a link between ERS and endothelial dysfunction in patients undergoing cardiac surgery with CPB.

## Limitations

Our work has several major limitations. First, it is a pilot physiological study with a limited number of patients. We included patients with several types of surgery (valve and/or CABG) which could lead to a heterogeneity of the studied population. For example, it is known that valve surgery induces more systemic inflammation than coronary bypass surgery (36). It is therefore possible that ERS may be more pronounced in patients with valve surgery. Second, our work involved gene expression in the whole blood. As some of the proteins studied in our work cannot be measured in blood without complex cell isolation techniques, RNA quantification appeared to be the best compromise. RNA expression on whole blood measured using Paxgen tubes is strongly correlated with RNA expression in circulating leucocytes, we can thus assume that we detected variations in leucocyte gene expression (37). As it is known that UPR plays a crucial role in immune cells, including differentiation, immune activation, antibody production and cytokine expression, it seemed relevant to study the leucocyte expression of UPR genes (38, 39). However, we may have missed a potentially greater variation in gene expression in tissues and organs, as observed in animal models (14, 15). Third, we performed the first sampling after anesthetic induction. It is known that propofol has a mild inhibitory effect on ERS several hours after induction (40). Given the mechanisms involved in UPR activation (gene transcription, protein translation), it is unlikely that there would be significant variations in UPR between pre-induction period and immediate post-induction period (a few minutes). Furthermore, since all patients had a standardized anesthesia protocol, it is likely that the effects of propofol on ERS were identical for all patients. Fourth, our samples were taken at only two post-operative timepoints and it is possible that we were not able to highlight the real peak of UPR expression. Moreover, our data show that the expression of several genes involved in UPR remains high 24 h after CPB compared to baseline. Our work did not allow us to conclude when the genes involved returned to baseline expression. Fifth, we only studied patients with cardiac pathologies, some of which are associated with ERS-inducing pathologies: diabetes, atherosclerosis, obesity, metabolic syndrome (41). It is therefore not certain that GRP78 and UPR kinetics would be identical in a population without pathologies. However, it should be noted that the GRP78 plasma levels found before CPB and 24 h after CPB were very close to those recently described in a group of healthy volunteers, which probably makes our results quite extrapolable to other populations (42). Finally, we defined organ failure according to usual clinical criteria, but we did not use a standardized organ failure score such as the Sequential Organ Failure Assessment score. This complicates the interpretation of the results and makes it more difficult to compare our results with those of other works. Further studies will need to be done using this type of score to define organ failure.

## Conclusion

We describe for the first time the kinetics of all UPR pathways during SIRS induced by cardiac surgery with CPB. We found that the plasma level of GRP78 was decreased at the initial phase of CPB-induced SIRS and that low relative GRP78 levels appeared associated with postoperative organ failure. However, further studies are needed to better understand ERS and UPR implications during systemic inflammation and acute organ failure in humans.

## Data Availability Statement

The raw data supporting the conclusions of this article will be made available by the authors, without undue reservation.

## Ethics Statement

The studies involving human participants were reviewed and approved by CPP Sud-Méditerranée II - Hôpital Sainte Marguerite - Pavillon 9 - 270 Boulevard Sainte Marguerite 13274 MARSEILLE. The patients/participants provided their written informed consent to participate in this study.

## Author Contributions

TC was involved in the study conception and design, in acquisition of data, in analysis and interpretation of data and in manuscript draft. ZD was involved in acquisition of data, in analysis and interpretation of data and in manuscript draft. XS was involved in acquisition and in interpretation of data. CT was involved in statistical analysis and in interpretation of data. EB, JS, and FD were involved in the study conception and design, in interpretation of data and in manuscript revision. VR, BV, and FT were involved in the study conception and design, in analysis and interpretation of data and in manuscript revision. All authors contributed to manuscript revision, read and approved the submitted version.

## Conflict of Interest

The authors declare that the research was conducted in the absence of any commercial or financial relationships that could be construed as a potential conflict of interest.

## Abbreviations

4PBA: 4-phenylbutyric acid
AKI: acute kidney injury
ATF: Activating Transcription Factor
CABG: coronary artery bypass grafting
CHOP: CCAAT/enhancer binding protein homologous protein
CPB: cardio-pulmonary bypass
Ct: cycle threshold
DNA: deoxyribonucleic acid
ER: endoplasmic reticulum
ERS: endoplasmic reticulum stress
GRP78: 78 kDa Glucose-Regulated Protein
HSPA5: Heat Shock 70kDa Protein 5
ICU: intensive care unit
IL: interleukin
IRE1α: Inositol-Requiring Protein-1 alpha
PERK: Protein Kinase RNA-like ER kinase
Pre-CPB: before cardio-pulmonary bypass
qPCR: quantitative polymerase chain reaction
RNA: ribonucleic acid
SAPS II: Simplified Acute Physiology Score II
SDHA: succinate dehydrogenase complex flavoprotein subunit A
SIRS: systemic inflammatory response syndrome
sXBP1: X-box binding protein 1
UPR: unfolded protein response
VCAM-1: Vascular Cell Adhesion Protein 1.
