# Supplementary figures and images for: A Weak Response to Endoplasmic Reticulum Stress Is Associated With Postoperative Organ Failure in Patients Undergoing Cardiac Surgery With Cardiopulmonary Bypass
Source: Front Med (Lausanne). 2021 Feb 15;7:613518. doi: 10.3389/fmed.2020.613518 (PMC7917111; doi:10.3389/fmed.2020.613518)

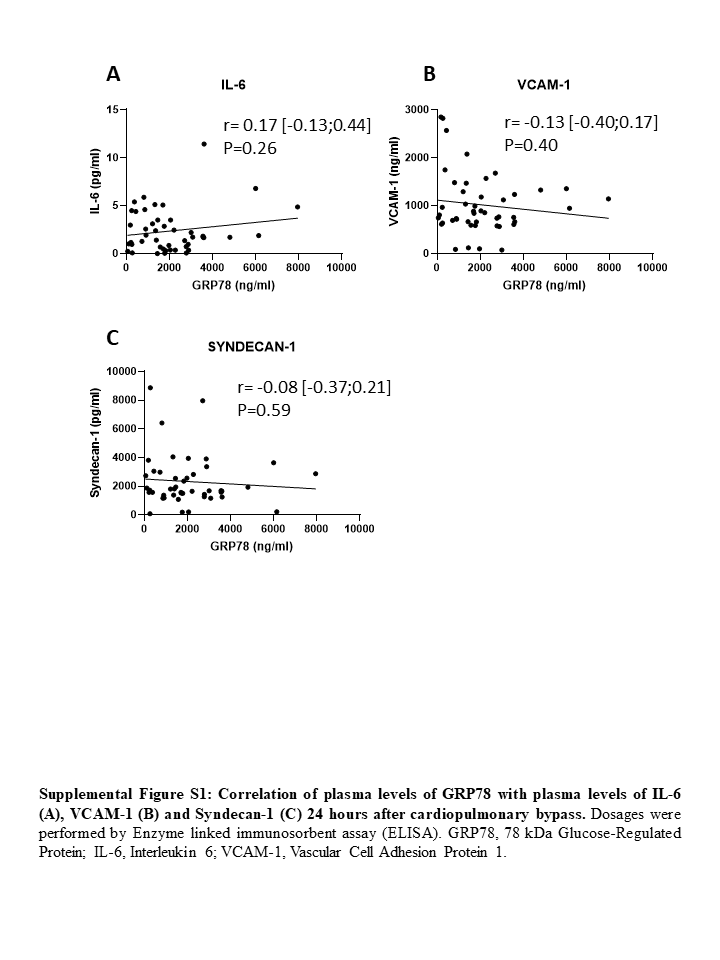

Supplement: Supplementary file 1 [file Image_1.TIF]
