# Supplementary material for: A Weak Response to Endoplasmic Reticulum Stress Is Associated With Postoperative Organ Failure in Patients Undergoing Cardiac Surgery With Cardiopulmonary Bypass
Source: Front Med (Lausanne). 2021 Feb 15;7:613518. doi: 10.3389/fmed.2020.613518 (PMC7917111; doi:10.3389/fmed.2020.613518)
Supplement: Supplementary file 2 [file Table_1.DOCX]

**Supplemental Table 1: GRP78 levels according to the type of organ failure in patients with and without organ failure 24 hours after cardiopulmonary bypass (CPB).**

|  | **Organ failure** | | |
| --- | --- | --- | --- |
| **GRP78 H24-CPB (ng/ml)** | **Hemodynamic failure requiring catecholamines** | **Invasive mechanical ventilation** | **Acute kidney injury** |
| 909 | 1 | 0 | 0 |
| 3557 | 0 | 1 | 0 |
| 1319 | 1 | 1 | 0 |
| 1571 | 1 | 0 | 0 |
| 1822 | 1 | 0 | 1 |
| 806 | 1 | 1 | 1 |
| 1436 | 1 | 0 | 0 |
| 1385 | 1 | 1 | 0 |
| 2427 | 1 | 0 | 0 |
| 3702 | 1 | 0 | 0 |

1, presence of organ failure; 0, absence of organ failure. GRP78, 78 kDa Glucose-Regulated Protein

**Supplemental Table 2:** **Comparison of basal level of GRP78 and Unfolded Protein Response gene expression (before cardiopulmonary bypass (CPB)) in patients with and without organ failure 24 hours after CPB.**

|  | **No Organ Failure (n=36)** | **Organ Failure (n=10)** | **p** |
| --- | --- | --- | --- |
| GRP78 (ng/ml) | 2348 [1638-3223] | 2952 [1968-4214] | 0.36 |
| *CHOP* | 1.0 [0.8-1.1] | 0.9 [0.7-1.2] | 0.66 |
| *ATF6* | 1.0 [0.7-1.1] | 1.1 [0.8-1.3] | 0.66 |
| *ATF4* | 1.0 [0.7-1.1] | 1.2 [0.9-1.4] | 0.07 |
| *HSPA5* | 1.0 [0.8-1.3] | 1.0 [0.9-1.2] | 0.88 |
| *sXBP1* | 1.0 [0.7-1.2] | 1.2 [0.9-1.2] | 0.49 |

Data are presented as median with interquartile range. ATF, Activating Transcription Factor; CHOP, CCAAT/enhancer binding protein homologous protein; GRP78, 78 kDa Glucose-Regulated Protein; HSPA5: Heat Shock 70kDa Protein 5; sXBP1, spliced RNA of X-box binding protein 1.
